# A guide to plant breeding for animal breeders

**DOI:** 10.1093/g3journal/jkag059

**Published:** 2026-03-31

**Authors:** Miguel Pérez-Enciso, Rex Bernardo

**Affiliations:** Centre for Research in Agricultural Genomics (CRAG), CSIC-IRTA-UAB-UB, Campus UAB, Bellaterra, Barcelona 08193, Spain; Institució Catalana de Recerca i Estudis Avançats (ICREA), Barcelona 08010, Spain; Department of Agronomy and Plant Genetics, University of Minnesota Twin Cities, Saint Paul, MN 55108, United States

**Keywords:** animal breeding, artificial intelligence, genomic prediction, genotype × environment interaction, plant breeding, teaching

## Abstract

Over the years, some animal breeding PhD graduates have found employment in major plant breeding companies, but this shift is often accompanied by culture shock. Animal breeding expertise, particularly in genomic prediction and mixed-model analysis, is valuable but insufficient for a straightforward transition to plant breeding. We stress two main limitations of an animal breeders training from a plant breeding perspective, and both stem from the ability to replicate plant genotypes: design and analysis of field experiments, and genotype by environment interaction. Besides genomic prediction and simulation, we argue that artificial intelligence and phenomics will further increase commonality between fields.

## Teaser

Over the years, some animal breeding Ph.D. graduates have found employment in major plant breeding companies, but this shift is often accompanied by culture shock. Animal breeding expertise, particularly in genomic prediction and mixed-model analysis, is valuable but insufficient for a straightforward transition to plant breeding. Pérez-Enciso and Bernardo stress two main limitations of an animal breeder’s training from a plant breeding perspective, and both stem from the ability to replicate plant genotypes: design and analysis of field experiments, and genotype by environment interaction. Besides genomic prediction and simulation, the authors argue that artificial intelligence and phenomics will further increase commonality between fields.

## Introduction

Although rooted in the same genetic principles, plant and animal breeding are operationally different. For decades, both disciplines evolved separately and have a scarcity of shared statistical software, journals that cover both fields, and joint scientific meetings. Animal breeding has relied heavily on best linear unbiased prediction since Henderson's seminal work in the 1960s to 1970s (Henderson 1975, 1984) with massive amounts of data from production-level herds. In contrast, plant breeding has for the most part emphasized phenotyping from carefully designed experiments in multiple environments, and BLUP was routinely adopted in plants only decades later with the development of genomic BLUP (GBLUP) by Bernardo (1994). While animal breeding has dealt with prediction in a scenario where uncertainty is due to scarce genetic information, plant breeding has traditional focused on estimating varietal performance wherein the risk stems from environmental unpredictability.

Over the years, some PhD graduates in animal breeding have moved to careers in major plant breeding companies, e.g. the first author of this article is an animal breeder who had been employed by a plant breeding company. In contrast, far fewer (if any) plant breeding Ph.D. graduates have moved to animal breeding, and we assume that this one-way movement is due to more private-sector job opportunities in plants. Animal breeders who have moved to plant breeding have often faced a steep learning curve because of key differences between breeding plants vs animals. Furthermore, a lack of understanding of each other's fields has limited the sharing of ideas between plant and animal breeders.

Our goal in this Dialogue and Debate is to highlight similarities and key differences between animal and plant breeding to enrich dialogue and facilitate transitioning from animal to plant breeding.

## Plant breeding in a nutshell

**Fig. 1.**
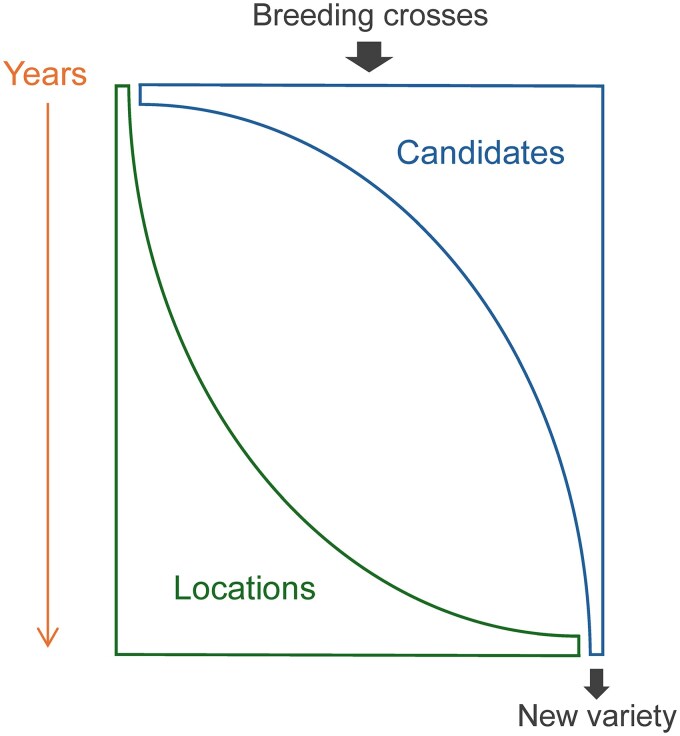
Survival of the fittest in a typical plant breeding program. Many candidates (lines, hybrids, or clones) are developed from controlled breeding crosses, and these candidates are evaluated at only a few locations during the first year. The best candidates are selected and re-evaluated in subsequent years, with progressively fewer candidates being evaluated at larger numbers of locations in subsequent years, until one or more new varieties are released.

Plant breeding methods depend on the modes of pollination and reproduction. Here we describe the three main breeding scenarios in plants. Regardless of the modes of pollination and reproduction, plant breeding involves (i) the development of experimental progeny and (ii) evaluation of the progeny in successive field tests of increasing stringency (Fig. 1).

Some species, such as soybean (*Glycine max* [L.] Merr.) and wheat (*Triticum aestivum* L.), are naturally self-pollinated and suffer from little or no inbreeding depression. A plant variety is then a homozygous line. Multiple pairs of parents are crossed and, from each cross, dozens to hundreds of partially homozygous and fully homozygous progeny are developed by 5 to 7 generations of self-pollination.

Other species such as maize (*Zea mays* L.) are naturally cross-pollinated but can also be self-pollinated. Inbreeding depression is severe which also means that hybrid vigor or heterosis is substantial. In maize, a plant variety is the cross between two fully homozygous lines (i.e. single-cross hybrid). Maize homozygous lines are most often developed by inducing haploids then doubling their chromosomes to create doubled haploids. Maize breeding is akin to crossbreeding in animals, as the two parents of a single-cross hybrid belong to two different genetic groups or backgrounds. However, productivity traits in maize are measured only on the crossbred individuals and are not routinely measured on the homozygous parents.

Species such as apple (*Malus domestica*) and potato (*Solanum tuberosum* L.) produce seeds but are also asexually propagated. A plant variety is a single individual (clone) that is propagated *en masse* by vegetative means.

Regardless of the species, the experimental progeny undergo a rigorous multi-year evaluation scheme in which many candidates are first evaluated in a limited number of locations (Fig. 1). The field evaluations are repeated in succeeding years, with an increasing number of locations as fewer candidates remain. To illustrate, a commercial soybean breeder might typically (i) make 200 crosses each year, (ii) develop 70,000 partially inbred lines across all 200 crosses, (iii) evaluate the lines in different numbers of locations for five years (e.g. 1 to 2 locations in year 1 and 20 to 50 locations in year 5), and (iv) release 0 to 5 homozygous lines as new plant varieties (Bernardo 2023).

Many tools and technologies are used throughout the breeding process. Animal breeders are familiar with some of these approaches, such as genomic prediction and simulation to guide breeding strategies and resource allocations. Animal breeders, however, are unfamiliar with many tools used in plant breeding. These include GPS-guided precision planters to ensure planting uniformity; drones to record data and pinpoint problematic spots (e.g. poor germination) via image analysis in a field experiment; statistical analyses to correct for local field heterogeneity; on-site climate data recorders for envirotyping; crop growth modeling of genotype × environment interaction for proper geographic placement of plant varieties; and continuous indoor or outdoor nurseries to speed up the breeding process.

## Key biological differences that influence how plants vs animals are bred

### Plants can be genetically replicated but animals cannot

This difference has the largest impact on how plants vs animals are bred. As described above, several mechanisms allow plants to be replicated. Homozygous lines and F_1_ hybrids can be multiplied easily. Asexual propagation leads to clones of individuals regardless of their level of heterozygosity.

In contrast, animals cannot be genetically replicated and controlled experiments to compare identical genotypes cannot be designed. Because animal breeders cannot increase the amount of information on a given candidate by sheer replication, they resort to information from relatives in genetic evaluation. These relatives are scattered across different farms, and genetic evaluations are based on massive amounts of unbalanced and messy production-level data rather than from randomized and replicated experiments.

For an animal breeder, heritability is a population-based parameter. In contrast, heritability in plants is expressed in multiple ways. Plant breeders refer to heritability on an individual-plant basis if measurements are taken on individual plants vs heritability on a genotype-mean basis if measurements are taken on multiple plants. Heritability of genotype-means can be arbitrarily increased with the number of replications. In plant breeding, heritability is therefore a function of both the population and the extent to which the population is phenotyped. Furthermore, plant breeders often do not express heritability in terms of the additive variance (*V*_A_). For example, the variance among recombinant inbreds is *V*_RI_ = 2*V*_A_. Plant breeders often calculate the heritability among means of recombinant inbreds as *V*_RI_/*V*_P_ = 2*V*_A_/*V*_P_, where *V*_P_ is the phenotypic variance.

In plants, improved genetic stock is in the form of plant varieties that are easily delivered to growers as seeds or vegetative propagules. Tissue culture also allows the multiplication of high-value plant species such as oil palm (*Elaeis guineensis* Jacq.). In animals, delivery of improved genetic stock occurs mostly via semen or embryos. The availability of either fresh or frozen semen technology is critical. While frozen semen in dairy cattle allows immediate dissemination of genetic progress worldwide, the use of fresh semen requires a multiplier nucleus and leads to a lag in genetic improvement.

### Animals can move but plants cannot

Professor A.E. Freeman at Iowa State University used to say, “*When a cow is thirsty, it goes to the water. But when a plant is thirsty, it stays thirsty*” (Bernardo 2020a). The inability of a plant to move leads to large amount of genotype × environment (G × E) interaction in plants.

The G × E interaction in plants leads to the need for field trials in multiple locations and years. For example, by the time a maize hybrid is released commercially, it would have been tested in hundreds of location-year combinations. Factors such as soil type and cultural management practices are largely stable from year to year, but plant breeders and growers do not know what next year's temperatures and precipitation patterns would be. The ability to genetically replicate plants allows screening of the same candidate for multiple abiotic stresses such as drought and low soil nitrogen, as well as multiple biotic stresses such as disease and insect pest resistance. Over the years, plant breeders have also developed multiple ways to study and assess G × E interaction, e.g. stability analysis, reaction norms, factor analysis, envirotyping, crop and physiological modeling, satellite imagery. These sophisticated statistical tools are infrequently used in animal breeding.

With few exceptions (e.g. Silva Neto et al. 2024), G × E interaction has been traditionally ignored in animal breeding for several reasons. First, most livestock are reared indoors or in highly controlled environments, which minimizes G × E interaction. Second, even species under extensive management move around often to minimize outdoor harshness, e.g. looking for shade or water. A third, and perhaps the most relevant reason, is that the inability of genetically replicate an animal leads to a dearth of information to assess and model G × E interaction. Nevertheless, G × E interaction analysis tools such as extended factor analyses have been popularized by animal breeders (Meyer 2009) and there is a growing interest in tackling G × E interaction in animals (Murani et al. 2023).

## GBLUP, simulations, and future commonalities

Genomic prediction currently provides the strongest unity between plant and animal breeding (Hickey et al. 2017), even if the specific GBLUP models differ, e.g. animal model vs single-cross prediction. Although animals cannot be genetically replicated, random partial replication of an animal's genotype occurs via identity by descent across multiple relatives. Marker or sequence data likewise capture genetic information replicated across animals or plants. Genomic prediction is most relevant in the early stages of a plant breeding program, when plant and animal breeding are most similar: the amount of phenotypic information per candidate is null or very small and selection intensity is high. At later stages, genomic prediction in plant breeding becomes almost irrelevant because candidates are extensively phenotyped. Genetic replication at the marker or sequence level may allow tractable solutions to analyzing G × E interaction in animals, i.e. identifying Quantitative Trait Loci (QTL) associated with an increased G × E interaction (Chen et al. 2010; Xavier et al. 2025).

Breeding is becoming increasingly computer-based rather than theory-driven (Bernardo 2020b), as attested by simulation being important in both plant and animal breeding. Some software can be used in both scenarios (Pérez-Enciso et al. 2020; Gaynor et al. 2021), and this is an area where plant and animal breeders can easily cooperate. Examples include digital twins to assess current breeding strategies, allocate resources, and explore new breeding approaches (Neethirajan and Kemp 2021; Wang 2024). Specific software could be developed to capture subtle differences between plant and animal breeding.

We have previously argued that the current limitations of animal breeding are caused by phenotyping rather than genotyping (Pérez-Enciso and Steibel 2021). Plant breeders have long ago developed sensor-based technologies for phenotyping (Cobb et al. 2013; Araus and Cairns 2014), and we conjecture that sensor technology and artificial intelligence (AI) algorithms will become equally important in both plant and animal breeding fields. Certainly, there are key differences that we do not wish to oversimply: for instance, plant breeders are largely concerned with static images and environmental sensors, whereas animal breeders often analyze videos for behavior appraisal. Under the hood, however, many modern AI algorithms can be applied to highly heterogeneous tasks. In all, we expect then that AI, phenomics, simulation and genomic prediction will become shared commonalities that will facilitate the exchange of ideas and methods across disciplines.

## Easing a transition from animal to plant breeding

Animal breeding expertise, particularly in genomic prediction and mixed-model analysis, is insufficient for a straightforward transition from animal to plant breeding. We stress two main limitations of an animal breeder's training from a plant breeding perspective, and both stem from the ability to replicate plant genotypes: design and analysis of field experiments, and G × E interaction.

Experimental designs in plant breeding have evolved over the years. Plant breeding programs have largely shifted to having single-replication experiments at a given location so that the number of locations can be maximized, in accordance with statistical theory. This shift to single-replication designs has made classical experimental designs, such as randomized complete block designs or lattice designs, much less important today. However, field plot techniques to control within-location error remain important, and spatial correction for field heterogeneity has become much more important. The design and analysis of field experiments is probably the most difficult topic for a “pure” animal breeder. G × E interaction, in turn, is making its way into the animal breeding literature (Murani et al. 2023). As adaptation to climate change and concern for resource efficiency becomes more important, G × E analysis techniques should be adopted by animal breeding curricula. Nevertheless, the impact of including G × E interaction in animal genomic prediction will be smaller than in plants for the reasons discussed.

From a practical point of view, we see two ways to ease a transition from animal breeding to plant breeding. First, courses that attempt to unify both fields can be developed. Such courses would emphasize genomic prediction, mixed-model methodology, field experimentation, G × E interaction, simulation, and AI. One benefit is that plant breeding students will also gain knowledge of animal breeding. Joint courses would reveal not only key differences but also subtle differences, e.g. reasons for the infrequent use of formal selection indices in plant breeding (i.e. difficulty in specifying economic weights) and different meanings of the same term (e.g. maternal effects in plants referring to the effects of maternally transmitted genes in the chloroplast).

As we have discussed, however, unifying the 2 fields in a single curriculum is difficult because the biological, unavoidable differences between plants and animals have major impacts on breeding. A second, simpler way for animal breeding students to learn about basic plant breeding is using resources already at hand. This could entail taking a graduate course in plant breeding, self-teaching, e.g. Bernardo (2023), or doing an internship at a plant breeding company. The latter would allow a first-hand look into the inner workings of a plant breeding company, including company-specific jargon. Learning jargon is a major benefit, as the first author often found this the most limiting and confusing feature of plant breeding.
